# Brain-wide analysis reveals movement encoding structured across and within brain areas

**DOI:** 10.1038/s41593-025-02114-x

**Published:** 2025-11-18

**Authors:** Ziyue Aiden Wang, Balint Kurgyis, Susu Chen, Byungwoo Kang, Feng Chen, Yi Liu, Dave Liu, Karel Svoboda, Nuo Li, Shaul Druckmann

**Affiliations:** 1 Stanford University, Stanford, Palo Alto, CA USA; 2 Janelia Research Campus, Howard Hughes Medical Institute, Ashburn, VA USA; 3 Baylor College of Medicine, Houston, TX USA; 4 Allen Institute for Neural Dynamics, Seattle, WA USA; 5 Duke University, Durham, NC USA

**Keywords:** Motor control, Computational neuroscience

## Abstract

Movement-related activity has been detected across much of the brain, including sensory and motor regions. However, much remains unknown regarding the distribution of movement-related activity across brain regions, and how this activity relates to neural computation. Here we analyzed movement-related activity in brain-wide recordings of more than 50,000 neurons in mice performing a decision-making task. We used multiple machine learning methods to predict neural activity from videography and found that movement-related signals differed across areas, with stronger movement signals close to the motor periphery and in motor-associated subregions. Delineating activity that predicts or follows movement revealed fine-scale structure of sensory and motor encoding across and within brain areas. Through single-trial video-based predictions of behavior, we identified activity modulation by uninstructed movements and their impact on choice-related activity analysis. Our work provides a map of movement encoding across the brain and approaches for linking neural activity, uninstructed movements and decision-making.

## Abstract (web summary)

Using a large dataset of neural recordings and modern video-analysis methods, Wang, Kurgyis et al. describe a rich, structured encoding of movement across and within brain areas in mice performing both instructed and uninstructed movements in a task.

## Main

A standard view of the function of the nervous system is the translation of sensory inputs into action^1–3^. According to this view, the brain is parcellated into sensory and motor areas, with association areas in between^4^. On the other hand, decades of neurophysiological recordings have found activity related to movement throughout sensory and motor regions of the brain. For example, neurons in visual cortical areas are modulated by eye movement in primates^5,6^ and mice^7^; neurons in the barrel cortex are modulated by movement of the whiskers in rodents^8–10^; and activity related to licking, locomotion and other motor behaviors causes modulation of neural activity across multiple cortical regions^11–16^. Recent studies suggest that movement-related signals can account for a substantial proportion of ongoing neural activity across both sensory and motor areas^17–21^, with differing degrees across brain areas. However, these studies have examined a few brain areas at a time and different studies relied on diverse behaviors and recording methods. There has not been a comprehensive characterization of movement-related activity across many relevant brain areas in a single behavior. It thus remains unclear how movement-related activity is distributed across the brain and whether there are systematic differences between brain areas.

Multiple statistical methods have been proposed to relate ongoing movements and neural activity at the level of single neurons or neural populations^22–24^. Yet, existing methods do not distinguish between different types of movement-related encoding. For example, movement-related activity could reflect motor commands, efference copies, reafferent signals from sensory organs or mixtures of these signals^25^.

The presence of motor signals also raises the question of how these signals influence neural computations. In sensory cortical regions, movement-related activity can modulate sensory coding to enable active sensation^10,13^ and predictive coding^12,15^. But in other brain regions, including frontal cortex, the impact of movement-related activity on neural computation is not well understood. Many laboratory tasks require animals to perform instructed movements to report decisions, for example, pressing a lever. However, animals perform additional uninstructed movements, which can be correlated with the cognitive process under study^26^, such as small movements biased toward the direction of future choice as evidence is accumulated. Indeed, neural signals related to accumulated evidence have been reported in muscle tensions^27^ or even in ongoing movement execution^28,29^. Motor-related signals have been found in decision-making and motor planning areas of the brain^17,20,22^, but these studies did not distinguish between decision- and movement-related activity and compare different types of encoding across brain regions. Consequently, it remains unclear how pervasive uninstructed movement signals are across the brain, and how they are related to neural activity modulated by an animal’s decision.

To address these questions, we analyzed recordings of more than 50,000 neurons, recorded in more than a dozen cortical and subcortical structures, simultaneously with high-speed video of orofacial movements, while mice performed a decision-making task^30,31^. We tested multiple methods to solve the computational problem of relating two high-dimensional, complex time-series datasets: pixels of behavioral videos describing the movement of the animal and time-varying spike rates of neurons recorded in specific brain regions. Although movement-related signals were widespread, the strength of movement-related signals differed across areas and across subdivisions of areas, with stronger movement signals in motor areas and motor subdivisions. The high temporal resolution of electrophysiology enabled us to distinguish activity predicting versus following movement, parsing putative sensory- and motor-related signals within brain areas. Further, we distinguish between neurons whose modulation is primarily movement-dependent versus others whose modulation is more task-contingency-dependent. The prevalence of these two types of neurons systematically differed across brain areas, with enriched task-contingency modulation in forebrain and midbrain regions. Our study offers a principled approach to dissecting the relationship of movements and cognition across multiregional neural circuits.

## Results

### Neural activity explained by movement differs across brain areas

**Fig. 1:**
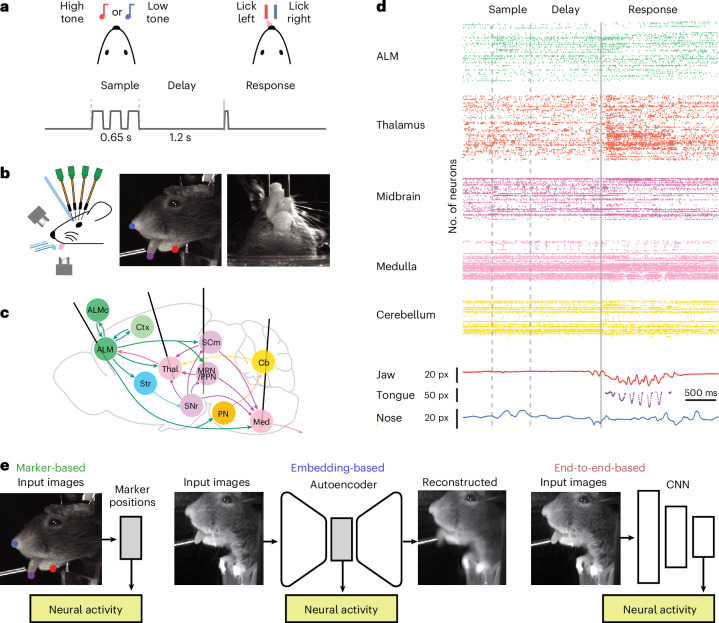
Multiregional neural recordings and prediction of neural activity from video. **a**, Delayed response task. **b**, Simultaneous video and neural recording. **c**, Example recording configuration. **d**, Raster plot of recorded neurons (top) and traces of body part marker locations for a single trial. **e**, Three approaches to predict neural activity from video. Left: marker-based analysis. For each video frame, each of the markers (jaw, nose or tongue) is a two-dimensional vector representing the vertical and horizontal positions. Middle: embedding-based analysis. For each frame, the embedding vector is a 16-dimensional vector. Right: end-to-end learning with deep neural network. ALMc, contralateral ALM; Cb, cerebellum; CNN, convolutional neural network; Ctx, cortex; Med, medulla; MRN, midbrain reticular nucleus; PN, pons; PPN, pedunculopontine nucleus; px, pixels; SCm, superior colliculus, motor related; SNr, substantia nigra, reticular part; Str, striatum; Thal, thalamus.

Populations of individual units were recorded while mice performed a memory-guided movement task (Fig. 1). Mice were trained to perform directional licking (lick-left or lick-right) depending on the frequency of a series of pure tones presented to the animal (12-kHz tones instruct lick-left; 3-kHz tones instruct lick-right) to obtain water rewards^32^ (Fig. 1a). In between the stimulus delivery and the behavioral response, mice were required to withhold licking for 1.2 s. We refer to the time period in which the sensory stimulus is presented as the ‘sample’ epoch, the period in which mice were required to respond as the ‘response’ epoch and the period in between as the ‘delay’ epoch. High-speed (300 Hz) videos of the face and paws were acquired from side and bottom views, together with neural population recordings (Fig. 1b). Two to five Neuropixels^33^ probes were used simultaneously to record extracellular activity in multiple regions of the mouse brain, including anterolateral motor cortex (ALM), an area critical for directional licking decisions^3,34–36^, as well as medulla, midbrain, striatum and thalamus, which form multiregional networks with ALM (Fig. 1b–d). Recording locations were registered to the Allen Common Coordinate Framework (CCF, v.3) and thus mapped^37^ to the Allen Reference Atlas^38^.

To examine the relationship of neural activity and ongoing movements, we analyzed facial and paw movements during task performance using three approaches. First, in the marker-based approach (Fig. 1e), we marked the nose, tongue and jaw in training data. DeepLabCut^24^ was used to track the two-dimensional location of the three markers in each video frame. We then regressed neural activity based on the time-series of the marker positions (Fig. 1e, left). Second, in the embedding approach, we used autoencoders^22^ to learn a low-dimensional embedding of the videos. The autoencoders reconstructed each frame through a low-dimensional bottleneck (Fig. 1e, middle). The encoder was a convolutional neural network, and the decoder was linear (Methods). In this architecture each frame was transformed into a 16-dimensional vector and the time-series of this 16-dimensional vector was then used to predict neural activity. Third, in the end-to-end learning approach we trained neural networks to directly predict neural activity from video (Fig. 1e, right, and Methods). The marker-based approach was the least expressive, as we manually selected a small number of features. The embedding approach was more expressive in that the nonlinear encoder network could learn a richer, if still low-dimensional, representation. The end-to-end approach was the most expressive as it could make full use of the high-dimensional dynamics in the video to explain activity.

**Fig. 2:**
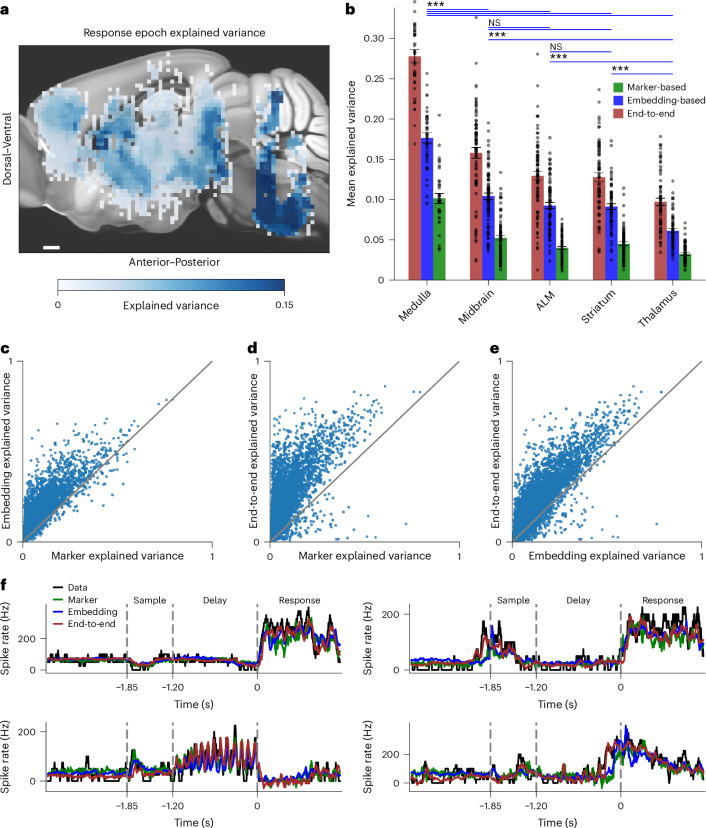
Movement encoding varies across brain areas. **a**, A two-dimensional spatial map of brain-wide prediction of neural activity from video. Each voxel is 150 × 150 µm^2^ in the sagittal plane and spans the brain in the third dimension. Color corresponds to mean variance explained by the embedding-based pipeline over all the neurons contained within each voxel. For visualization a 3 × 3-voxel median filter was applied on the heatmap. Scale bar, 0.5 mm. **b**, Performance of video-based prediction with neurons pooled according to brain area. Error bars correspond to the s.e.m. of insertion-averaged values. Overlaid markers correspond to individual insertions; numbers of insertions are: medulla *n* = 36 insertions, midbrain *n* = 79 insertions, ALM *n* = 77 insertions, striatum *n* = 67 insertions, thalamus *n* = 62. Visual representation of statistics corresponds to pairwise comparison for the embedding-based predictions with two-sided Mann–Whitney *U* test and Bonferroni correction, ***P < 0.001, NS *P* > 0.05; for exact *P* values see Extended Data Fig. 2. **c**, Comparison of single-neuron explained variance between marker-based method (*x* axis) and embedding-based method (*y* axis). Each dot corresponds to a neuron. **d**, Comparison of single-neuron explained variance between marker-based method (*x* axis) and end-to-end learning (*y* axis). Each dot corresponds to a neuron. **e**, Comparison of single-neuron explained variance between embedding-based method (*x* axis) and end-to-end learning (*y* axis). Each dot corresponds to a neuron. Session-averaged improvements in explained variance are, for embedding-based approach versus marker-based approach, mean improvement = 155 ± 5% s.e.m., *n* = 105 sessions, *P* < 0.001; end-to-end learning versus marker-based approach, mean improvement = 330 ± 9% s.e.m., *n* = 105 sessions, *P* < 0.001; end-to-end learning versus embedding-based approach, mean improvement = 76 ± 3% s.e.m., *n* = 105 sessions, *P* < 0.001, one-sided Wilcoxon signed-tank test with Bonferroni correction. **f**, Spike rates of four example neurons during four single trials. Spike rates are plotted (black line) overlaid with their prediction from the marker-based (green), embedding-based (blue) and end-to-end (brown) pipelines. Note, negative spike rate could have been removed post hoc but here we show raw prediction output. The explained variances for the four neurons are the following: from top left to bottom right: medulla neuron, explained variance marker 0.44, embedding 0.68, end-to-end 0.83; medulla neuron, explained variance marker 0.17, embedding 0.36, end-to-end 0.39; medulla neuron, explained variance marker 0.13, embedding 0.23, end-to-end 0.26; medulla neuron, explained variance marker 0.22, embedding 0.25, end-to-end 0.46.

Our analysis recapitulated the finding that movement-related activity is widely distributed across the brain (Fig. 2a,b). However, our analysis also revealed clear differences across brain regions in the ability of the video recordings to predict neural activity (Fig. 2a,b). For example, explained variance was especially high in the medulla (Fig. 2a). Variance explained in the medulla was significantly larger than midbrain (medulla explained variance from the embedding method 0.176 ± 0.06 s.e.m., *n* = 36 insertions compared with 0.104 ± 0.004, *n* = 79 of midbrain, *P* < 0.001, Mann–Whitney test, note insertions rather than sessions were used since in some sessions recordings were performed for a given brain area simultaneously across two hemispheres). Explained variance followed a logical progression, with greater explained variance in areas closer to the sensory or motor periphery (Fig. 2b).

**Extended Data Fig. 1:**
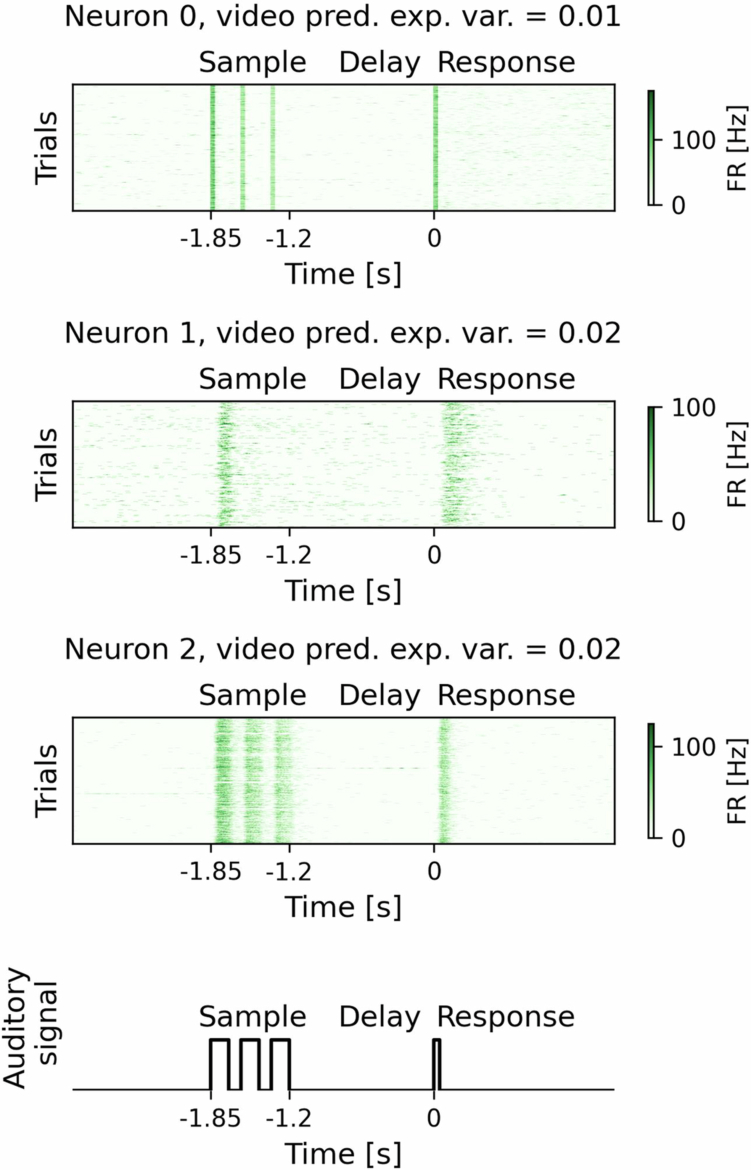
Neurons with reliable responses but whose activity is not well predicted from video. Heatmap of firing rate as a function of time. Each row corresponds to a single trial. Top neuron is a midbrain neuron, middle neuron is a striatum neuron, bottom neuron is a thalamic neuron. Bottom panel shows schematic for the presented auditory cues.

**Extended Data Fig. 2:**
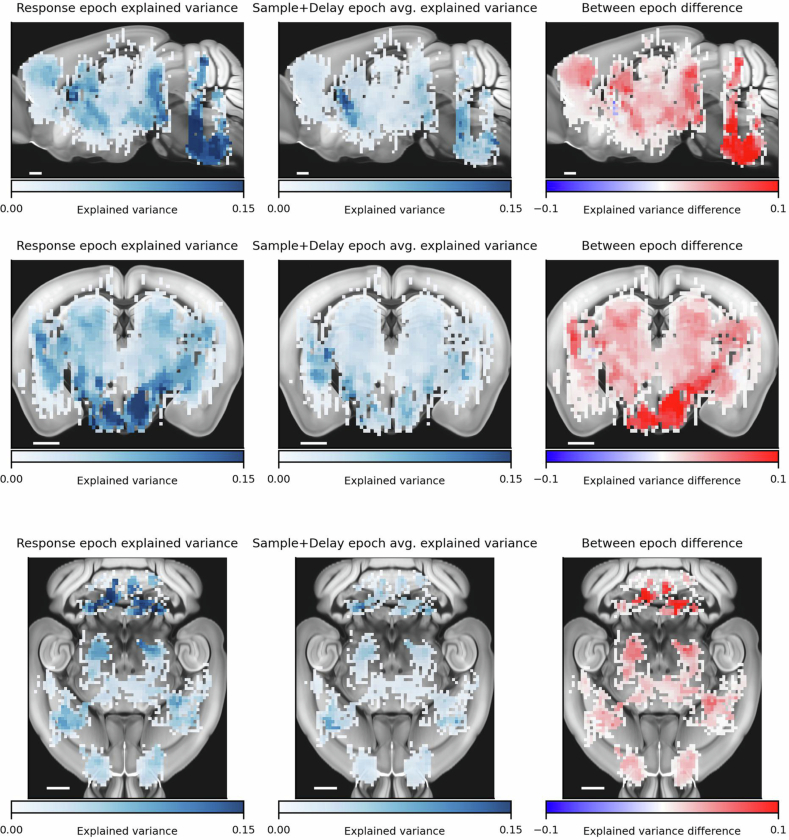
Voxel level explained variance separately per epoch. Response epoch with rich licking movements (left column) sample/delay epochs (middle column) and difference between the response to sample/delay epochs (right column). The rows correspond to different projections: sagittal on top, coronal in the middle and horizontal on the bottom. Voxel size is 150 µm squared in the respective projection plane and spans the brain in the third direction. Color corresponds to average of single neurons’ explained variance within each voxel (left and middle columns) and to the difference between response epoch and sample/delay epoch explained variance (right column). For visualization a 3x3 median filter was applied to the heatmaps.

**Extended Data Fig. 3:**
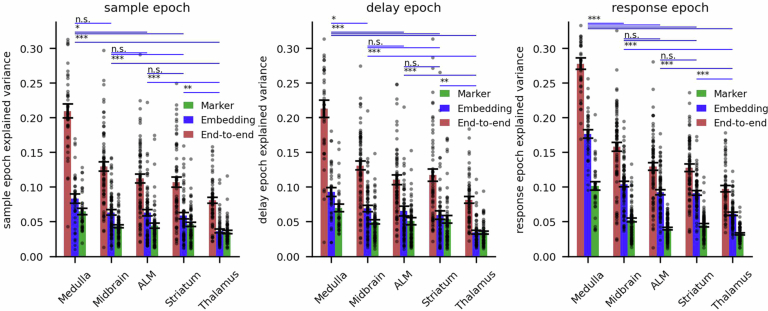
Explained variance by brain area across methods and epochs. Single neuron explained variances are pooled by anatomical regions. Error bars represent standard error of insertion averaged means. Overlaid markers show individual mean values for individual insertions. Visual representation of statistics corresponds to pairwise comparison for the embedding based predictions with two-tailed Mann-Whitney U test and Bonferroni correction, *** is p < 0.001, ** is p < 0.01, * is p < 0.05 and ns is p > 0.05. Sample epoch insertion averaged comparisons for embedding based method: Medulla (0.0833 ± 0.0066, n = 36) vs Midbrain (0.0632 ± 0.0039, n = 70), p = 0.0785; Medulla (0.0833 ± 0.0066, n = 36) vs ALM (0.0629 ± 0.0047, n = 69), p = 0.0382; Medulla (0.0833 ± 0.0066, n = 36) vs Striatum (0.0586 ± 0.0046, n = 59), p = 0.0212; Medulla (0.0833 ± 0.0066, n = 36) vs Thalamus (0.0364 ± 0.0027, n = 57), p = 3.77e-07; Midbrain (0.0632 ± 0.0039, n = 70) vs ALM (0.0629 ± 0.0047, n = 69), p = 1; Midbrain (0.0632 ± 0.0039, n = 70) vs Striatum (0.0586 ± 0.0046, n = 59), p = 1; Midbrain (0.0632 ± 0.0039, n = 70) vs Thalamus (0.0364 ± 0.0027, n = 57), p = 3.45e-06; ALM (0.0629 ± 0.0047, n = 69) vs Striatum (0.0586 ± 0.0046, n = 59), p = 1; ALM (0.0629 ± 0.0047, n = 69) vs Thalamus (0.0364 ± 0.0027, n = 57), p = 2.19e-04; Striatum (0.0586 ± 0.0046, n = 59) vs Thalamus (0.0364 ± 0.0027, n = 57), p = 0.00256. Delay epoch insertion averaged comparisons for embedding based method: Medulla (0.0927 ± 0.0065, n = 36) vs Midbrain (0.0690 ± 0.0045, n = 67), p = 0.0304; Medulla (0.0927 ± 0.0065, n = 36) vs ALM (0.0654 ± 0.0069, n = 70), p = 6.91e-04; Medulla (0.0927 ± 0.0065, n = 36) vs Striatum (0.0594 ± 0.0063, n = 57), p = 1.81e-04; Medulla (0.0927 ± 0.0065, n = 36) vs Thalamus (0.0347 ± 0.0028, n = 53), p = 8.06e-10; Midbrain (0.0690 ± 0.0045, n = 67) vs ALM (0.0654 ± 0.0069, n = 70), p = 1; Midbrain (0.0690 ± 0.0045, n = 67) vs Striatum (0.0594 ± 0.0063, n = 57), p = 0.211; Midbrain (0.0690 ± 0.0045, n = 67) vs Thalamus (0.0347 ± 0.0028, n = 53), p = 1.15e-07; ALM (0.0654 ± 0.0069, n = 70) vs Striatum (0.0594 ± 0.0063, n = 57), p = 1; ALM (0.0654 ± 0.0069, n = 70) vs Thalamus (0.0347 ± 0.0028, n = 53), p = 4.24e-05; Striatum (0.0594 ± 0.0063, n = 57) vs Thalamus (0.0347 ± 0.0028, n = 53), p = 0.00741. Response epoch insertion averaged comparisons for embedding based method: Medulla (0.1764 ± 0.0061, n = 36) vs Midbrain (0.1043 ± 0.0040, n = 79), p = 1.04e-11; Medulla (0.1764 ± 0.0061, n = 36) vs ALM (0.0923 ± 0.0037, n = 77), p = 1.24e-13; Medulla (0.1764 ± 0.0061, n = 36) vs Striatum (0.0912 ± 0.0034, n = 67), p = 1.03e-13; Medulla (0.1764 ± 0.0061, n = 36) vs Thalamus (0.0611 ± 0.0026, n = 62), p = 3.31e-15; Midbrain (0.1043 ± 0.0040, n = 79) vs ALM (0.0923 ± 0.0037, n = 77), p = 0.314; Midbrain (0.1043 ± 0.0040, n = 79) vs Striatum (0.0912 ± 0.0034, n = 67), p = 0.0999; Midbrain (0.1043 ± 0.0040, n = 79) vs Thalamus (0.0611 ± 0.0026, n = 62), p = 2.47e-11; ALM (0.0923 ± 0.0037, n = 77) vs Striatum (0.0912 ± 0.0034, n = 67), p = 1; ALM (0.0923 ± 0.0037, n = 77) vs Thalamus (0.0611 ± 0.0026, n = 62), p = 2.80e-08; Striatum (0.0912 ± 0.0034, n = 67) vs Thalamus (0.0611 ± 0.0026, n = 62), p = 8.26e-09. All comparisons were done with two-tailed Mann-Whitney U test and Bonferroni correction.

The ordering of brain areas in terms of predictive power was preserved across approaches, despite the greater predictive power of the more expressive models (Fig. 2c–e, improvement in explained variance for embedding-based approach versus marker-based approach, improvement = 155 ± 5% s.e.m., *n* = 105 sessions, *P* < 0.001; end-to-end learning versus marker-based approach, improvement = 330 ± 9% s.e.m., *n* = 105 sessions, *P* < 0.001; end-to-end learning versus embedding-based approach, improvement = 76 ± 3% s.e.m., *n* = 105 sessions, *P* < 0.001, one-sided Wilcoxon signed-tank test with Bonferroni correction). Better predictive accuracy of the more expressive models was clear when visualizing single-trial predictions (Fig. 2f). Thus, even at timescales of tens of milliseconds, our dataset supports data-intensive models in learning meaningful features, surpassing less expressive models in predictive accuracy. However, not all neurons were predictable, even among neurons with highly reliable responses (see Methods for definition and Extended Data Fig. 1). The activity profile of some of these neurons suggests that they represented the auditory cues delivered in the task, which were not phase-locked to movements (Extended Data Fig. 1). Comparison across epochs suggested that a large portion of activity was correlated with licking and associated facial movements (Extended Data Fig. 2), but the relative differences in movement encoding between areas were preserved (Extended Data Fig. 3).

**Fig. 3:**
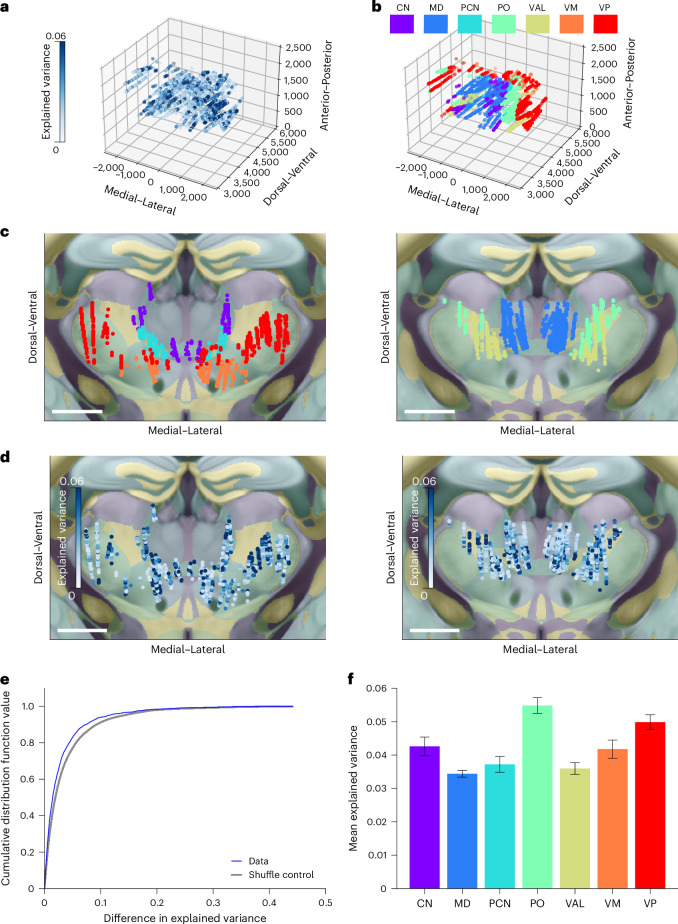
Differences of movement encoding across thalamic nuclei. **a**, Fraction of neural activity variance explained by video prediction. Each dot corresponds to a neuron. Color reflects variance explained by embedding-based prediction **b**, Annotations for thalamic nuclei. Each dot corresponds to a neuron. Neurons were mapped to their CCF coordinates and colored according to the annotation for that coordinate. **c**, Two-dimensional projection onto the coronal plane based on nuclei annotation along the full length of anterior–posterior axis. Each dot corresponds to a neuron. Color corresponds to nuclei annotation. Overlaid with high transparency is a map with color corresponding to Allen Reference Atlas annotation. Left plot shows more anterior portion of thalamus (AP = 6,600 µm); right plot shows more posterior (AP = 6,800 µm). Scale bar: 1 mm. **d**, Two-dimensional coronal projection of variance explained. Each dot corresponds to a neuron. Color reflects variance explained by embedding-based prediction. Left plot shows more anterior portion of thalamus; right plot shows more posterior. Overlaid with high transparency is a map with color corresponding to Allen Reference Atlas annotation. Scale bar: 1 mm. **e**, Variance explained varies nondiscontinuously across space. Plot shows cumulative distribution function of difference between variance explained of each neuron and its nearest-neighbor neuron. Data are in blue and 100 repetitions of neuron-by-neuron shuffle are in gray (Methods). **f**, Average explained variance across thalamic nuclei. Error bars correspond to s.e.m. Numbers of neurons in each region are: CN *n* = 235, MD *n* = 1,112, PCN *n* = 268, PO *n* = 600, VAL = 457, VM *n* = 221, VP *n* = 651. See Extended Data Fig. 4 for pairwise comparisons. CN, central lateral nucleus and central medial nucleus; MD, mediodorsal nucleus; PCN, paracentral nucleus.

**Extended Data Fig. 4:**
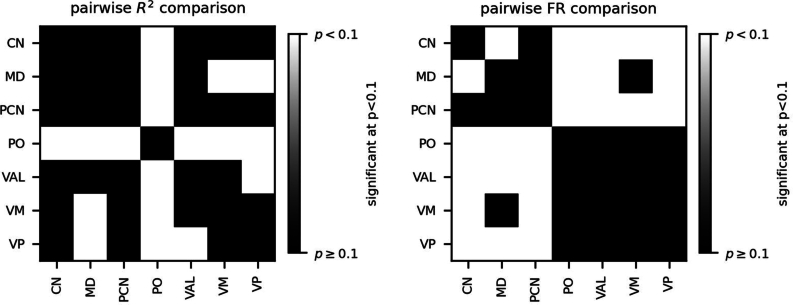
Pairwise comparisons between thalamic nuclei. The significance (with p < 0.1) of pairwise comparisons between thalamic nuclei are shown for explained variance (left) and firing rate (right). P-values are calculated with two-tailed Mann–Whitney U test and Bonferroni correction for multiple comparisons.

Registering neurons to the CCF allowed us to analyze encoding at the level of brain areas and subdomains, such as cortical layers and nuclei. As an example, our recordings sampled large portions of the anterior thalamus, which consists of multiple annotated nuclei. We found that explained variance was nonuniform across the thalamus (Fig. 3a–d, test against spatial uniformity, *P* < 0.0001, and Methods). We analyzed how variance explained changes over space by measuring the distribution of difference in variance explained between nearest-neighbor neurons. If variance explained changes as some smooth function over CCF space, then the difference in variance explained between a neuron and its nearest neighbors would be smaller than the difference between that neuron and a randomly selected one. We found that variance explained was significantly smaller between nearest-neighbor pairs, indicating spatially structured smooth changes in variance explained across thalamus (Fig. 3e, nearest-neighbor difference in explained variance smaller than shuffle control, *P* < 1 × 10^−3^). For additional analysis we grouped neurons into nuclei based on the Allen ontology^38^. For seven of the nuclei, we had a sufficient number of recorded neurons for analysis (threshold set at 100; see Methods and Supplementary Table 1 for definitions of nuclei). Explained variance based on the embedding method varied significantly among nuclei (Fig. 3c,d,f and see Extended Data Fig. 4 for pairwise statistics). Spike rate differences did not explain differences in variance explained (spike rate difference not significant between posterior complex (PO), ventral anterior–lateral complex (VAL), ventral medial nucleus (VM) and ventral posterior complex (VP), *P* > 0.1, yet variance explained differed significantly between VAL and PO/VM/VP, *P* < 1 × 10^−3^, one-way *F*-test between these four nuclei; also see Extended Data Fig. 4 for pairwise comparisons).

### Dissecting putative motor and sensory neural signals

**Fig. 4:**
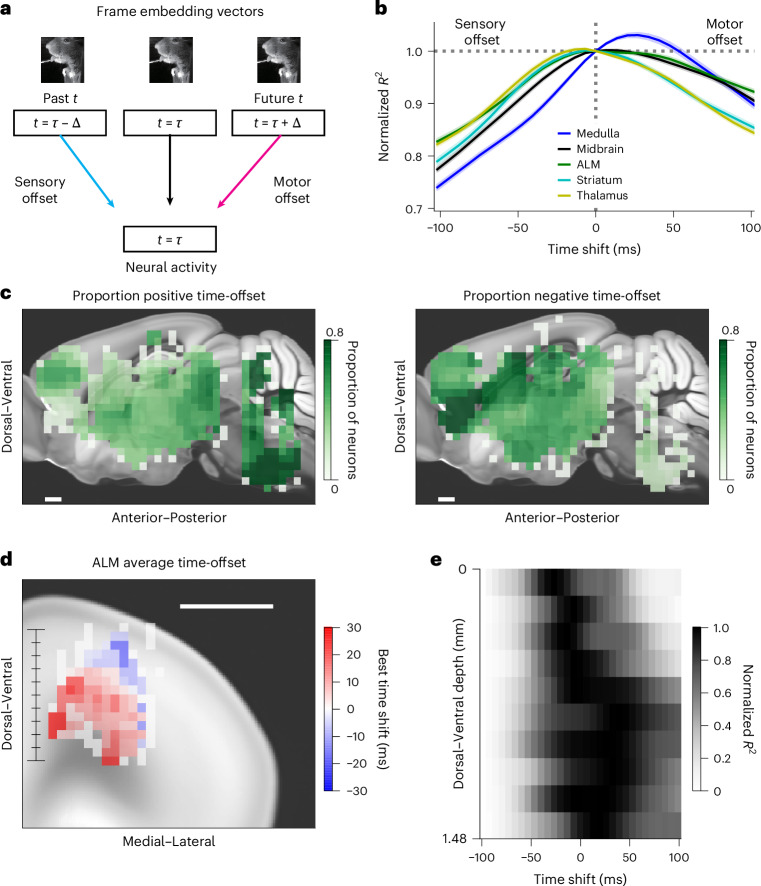
Temporal relation between neural activity and behavior. **a**, Schematic of video to activity prediction with different temporal offsets. Comparison across shifted time windows. **b**, Comparison of prediction accuracy across different temporal shifts averaged by brain area. For each area, the explained variance across different temporal shifts is shown as a line, normalized to the explained variance at zero temporal shift. Lines correspond to mean across sessions, colored by brain region; shaded area corresponds to the s.e.m. across sessions. **c**, Brain-wide spatial map of fraction of neurons with positive (left) and negative (right) best temporal offsets. Voxels are of size 300 µm squared in the sagittal plane and span the brain in the third direction. Color represents the proportion of neurons with positive time-offset (left) and negative time-offset (right) within each voxel. For visualization a 3 × 3-voxel median filter was applied on the heatmaps. Scale bar: 0.5 mm. **d**, Heatmap of best time-offsets for ALM neurons. Each voxel is 150 µm squared in the coronal plane and spans the brain in the third direction. Color corresponds to the average of best temporal offsets within that voxel. For visualization a 3 × 3-voxel median filter was applied on the heatmap. Scale bar: 1 mm. **e**, Comparison of explained variance across different temporal offsets as a function of cortical depth, taken as distance along the dorsal–ventral axis shown in **d**. Each row is normalized by its minimal and maximal values. Spearman rank correlation between cortical depth and best time-offset is *R* = 0.1, *P* = 0.0014, *n* = 1,059 neurons.

Movement-related signals could reflect motor commands, where activity is expected to lead movement. Alternatively, reafferent signals are expected to lag movement. The high temporal resolution of electrophysiology allowed us to analyze the temporal relation between neural activity and movement (Fig. 4). We shifted the window of video frames used to predict neural activity across a range of lead or lag times. We tested time windows both from the past and in the future relative to the analyzed neural activity (Fig. 4a). For a brain area involved in producing movement, the current activity predicts future movement, which will be reflected in future video frames. Thus, shifting the window of behavioral variables forward in time will yield better prediction (Fig. 4a). Conversely, if an area is sensory (for example, proprioceptive), then current activity follows past movements. Thus, shifting the window of behavioral variables backward in time will yield better prediction (Fig. 4a).

**Extended Data Fig. 5:**
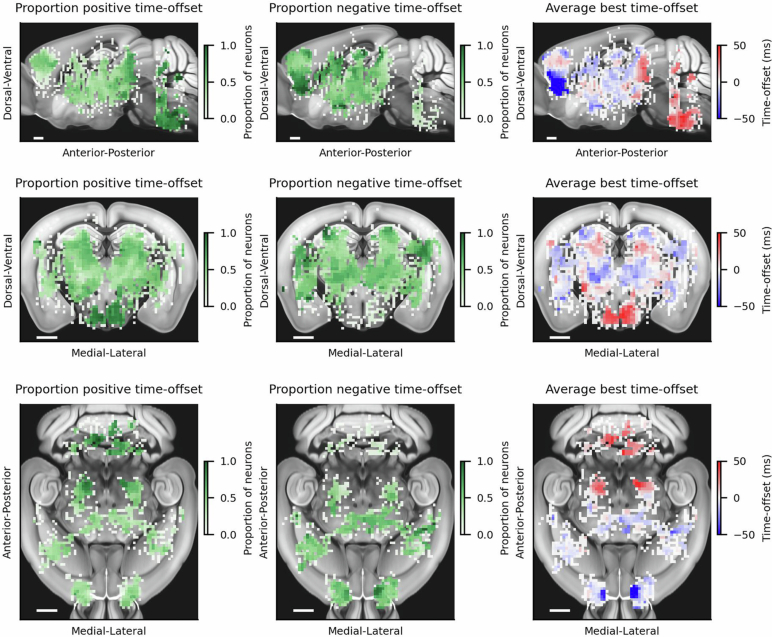
Spatial patterns in optimal video-activity time-offset. The heatmaps show the proportion of neurons with positive time-offset (left column), negative time-offset (middle column) and best time-offset (right column) in the sagittal plane (top row), coronal plane (middle row) and horizontal plane (bottom row). Each voxel is 150 µm squared in the respective plane and spans the brain in the third direction. Color corresponds to the proportion of neurons within each voxel with positive time-offset (left column), with positive time-offset (middle column) and to the average of single-neuron best time-offsets within each voxel (right column). For visualization a 3x3 voxel median filter was applied on the heatmaps.

**Extended Data Fig. 6:**
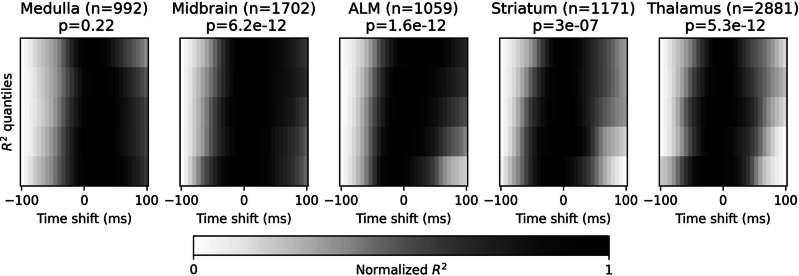
Relation between explained variance and video activity time offset. The neurons are first grouped into 5 quantiles based on their explained variance at zero offset, then the normalized explained variance as a function of time is averaged for neurons within each quantile. Each row is normalized by its maximum and minimum value. The p-values are calculated using Spearman rank correlation between explained variance and the best time-offset of neurons. Medulla n = 992 neurons, R = -0.06, p = 0.22; Midbrain n = 1702 neurons, R = 0.17, p = 6e-12; ALM n = 1059 neurons, R = 0.22, p = 2e-12; Striatum n = 1171 neurons, R = 0.15, p = 3e7; Thalamus n = 2881 neurons, R = 0.13, p = 5e-12; Spearman rank correlation with Bonferroni correction.

We performed this analysis in the response epoch, which had the strongest relation between movement and neural activity, using the embedding-based approach. We found clear differences across the brain (Fig. 4b,c), with differences both in the average optimal time-shifts (Extended Data Fig. 5) and in the proportion of neurons with positive versus negative time-shifts (Fig. 4c). A strong anterior–posterior pattern emerged, with neurons in the medulla having a strong preference for video-shifts into future time points that was also apparent when averaging neurons within brain regions and comparing across brain regions (Fig. 4b), consistent with the known role of medulla in controlling orofacial movement^39^. We observed that the best time-offset was positively correlated with the explained variance of the neurons (Extended Data Fig. 6) with the exception of medulla (Spearman rank correlation between best time-offset and explained variance of neurons not significant in medulla, *P* > 0.1 *n* = 992, and significantly positive in midbrain, *P* < 0.001, *n* = 1,702; ALM, *P* < 0.001, *n* = 1,059; striatum, *P* < 0.001, *n* = 1,171; thalamus, *P* < 0.001, *n* = 2,881, with Bonferroni correction). In other words, more motor-related neurons were better predicted by behavioral videos.

**Fig. 5:**
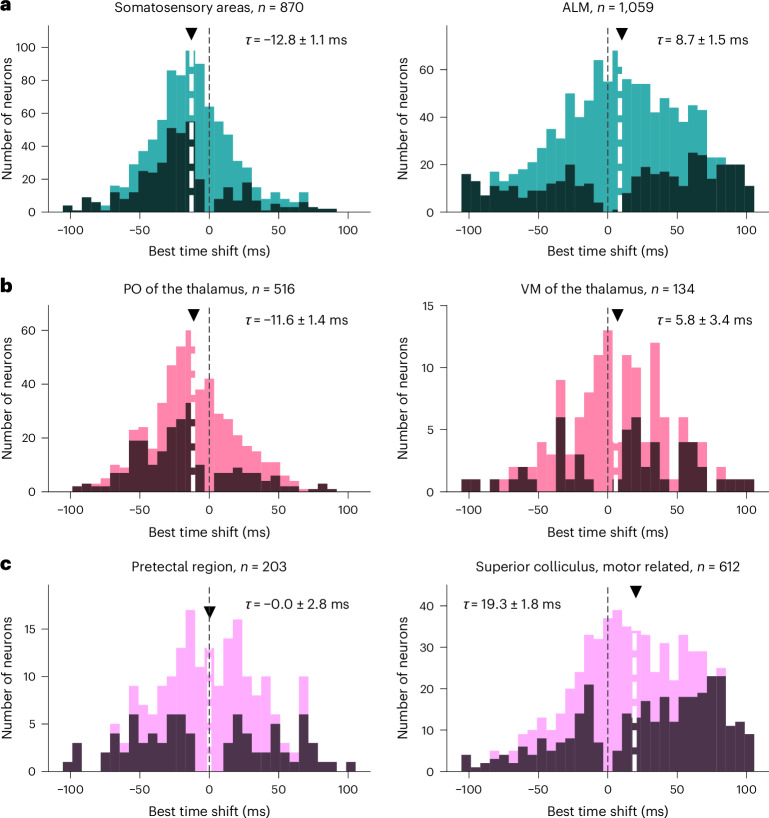
Temporal relation between activity and movement at the level of individual neurons. Histograms of optimal single-neuron time-offsets within each region. In each histogram darker colors indicate neurons with time-offset significantly different from zero. Black dotted line corresponds to zero time-offset and the black triangles and white dashed lines indicate mean; the numbers of neurons in the areas are noted in the subplot titles. The left column corresponds to more sensory-related areas and the right column more motor-related ones. **a**, Comparison between two cortical regions: somatosensory areas (left) and ALM (right). Somatosensory areas mean time-offset −12.8 ± 1.1 ms s.e.m., *n* = 870 neurons; ALM mean time-offset 8.7 ± 1.5 ms s.e.m., *n* = 1,059 neurons, *P* < 0.0001. **b**, Comparison between two thalamic subnuclei: PO of the thalamus (left) and VM of the thalamus (right). PO mean time-offset −11.6 ± 1.4 ms s.e.m., *n* = 516 neurons; VM mean time-offset 5.8 ± 3.4 ms s.e.m., *n* = 134 neurons, *P* < 0.001. **c**, Comparison between two midbrain subregions: pretectal region (left) and superior colliculus, motor-related (right). Pretectal region mean time-offset 0.0 ± 2.8 ms s.e.m., *n* = 203; superior colliculus mean time-offset 19.3 ± 1.8 ms s.e.m., *n* = 612, *P* < 0.001. The distributions were compared using one-sided Mann–Whitney *U* test and Bonferroni correction.

Neurons within each brain area had heterogeneous video-activity shift preference (Fig. 5), suggesting rich encoding of movement. Nevertheless, systematic differences were found between known structures associated with sensory versus motor functions. Among cortical regions, we found that somatosensory areas are more sensory tuned than ALM (Fig. 5a; somatosensory areas mean time-offset, −12.8 ± 1.1 ms s.e.m., *n* = 870 neurons; ALM mean time-offset, 8.7 ± 1.5 ms s.e.m., *n* = 1,059 neurons; the two distributions are different at *P* < 0.0001, one-sided Mann–Whitney *U* test), as expected. We additionally analyzed subregions within thalamus and midbrain and found significant differences in video time-offset. In thalamus (Fig. 5b), sensory thalamus PO followed movement (mean time-offset, −11.6 ± 1.4 ms s.e.m., *n* = 516 neurons) and motor thalamus VM led movement (mean time-offset, 5.8 ± 3.4 ms s.e.m., *n* = 134 neurons). In midbrain (Fig. 5c), we found a significant difference between pretectal region (mean time-offset, 0.0 ± 2.8 ms s.e.m., *n* = 203) and motor-related superior colliculus (mean time-offset, 19.3 ± 1.8 ms s.e.m., *n* = 612). Thus, we were able to uncover differences in sensory versus motor processing across subregions in subcortical structures (thalamus, *P* < 0.001; midbrain, *P* < 0.001, comparison of distributions with one-sided Mann–Whitney *U* test and Bonferroni correction). We also examined differences on a more fine-grained level in ALM (Fig. 4d,e). We found a change from more sensory-related signals to motor-associated signals as a function of cortical depth (best time-offset is significantly, positively correlated with cortical depth, *P* < 0.01, *n* = 1,059, Spearman rank correlation). This sensory-motor encoding is consistent with established anatomy of motor cortex, where superficial layers receive sensory signals, whereas deeper layers send motor signals to midbrain and medulla^34,35,40^.

### Analyzing and interpreting uninstructed movements

**Fig. 6:**
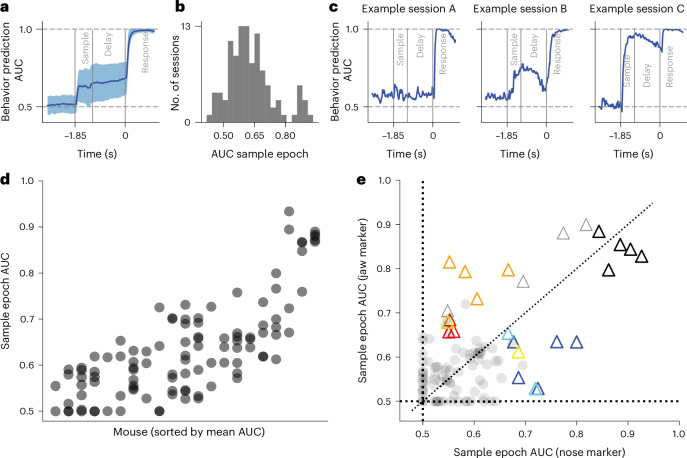
Prediction of single-trial behavior directly from video. **a**, Prediction accuracy of single-trial behavior from videos through embedding pipeline. Accuracy quantified through ROC AUC (*y* axis). Thick line indicates across session mean. Shaded area indicates standard deviation. **b**, Histogram of prediction accuracy during the sample epoch across sessions. **c**, Single sessions examples. Each panel corresponds to a single session, and each session is taken from a different mouse. **d**, Sample epoch prediction across all sessions and mice. Each *x*-axis location corresponds to an individual mouse. Each circle is a session. **e**, Prediction from single markers during the delay epoch. Each circle or triangle corresponds to a session. The *x*-axis value corresponds to ROC AUC from behavioral prediction using only the nose marker. The *y*-axis value corresponds to ROC AUC from behavioral prediction using only the jaw marker. To allow association of sessions to mice to be visible, only sessions that passed a high predictability criterion (AUC larger than 0.6) are shown as colored triangles. The rest are shown as circles. Colors of triangles correspond to individual mouse identity.

Are uninstructed movements related to decision-making? If uninstructed movements bear some relation to future choice-behaviors, then single-trial choices could be predictable directly from behavioral videos. We trained decoders to predict choice from behavioral videos (Fig. 6). We found that before the sample epoch, predictions of choice were at chance (area under the curve (AUC) of receiver operating characteristic (ROC) was 0.51 ± 0.06 s.d., *n* = 106 sessions, prediction based on embedding method; Methods), consistent with the lack of information regarding trial type at this point. In the sample and delay epochs, the mean AUC increased significantly (0.66 ± 0.12 s.d., *n* = 106 sessions, mean AUC ROC of the second half of sample epoch and the delay epoch). Thus, even before mice performed their explicit choice action (directional licking), uninstructed movements contained trial-type information. Soon after the go cue, prediction saturated at close to perfect performance (0.99 ± 0.01 s.d., *n* = 106 sessions, mean AUC ROC of the second half of response epoch), consistent with the choice being easily decodable from video of directional licking. Notably, predictive accuracy was highly variable across sessions in the sample and delay epochs (Fig. 6a–c). Future behavior was predictable from videos in some sessions, but not in others. In other words, animals were highly heterogenous in the extent they exhibited uninstructed, trial-type-related movements. Variability in predictability of actions from video was smaller within mice than across mice, consistent with the notion that individual animals adopted relatively consistent uninstructed movements (Fig. 6d, Calinski–Harabasz clustering score^41^ for clustering of within-animal points 10.45, compared with null model value of 1.02 ± 0.37 s.d.; higher scores correspond to stronger clustering, *P* < 0.001; Methods).

**Extended Data Fig. 7:**
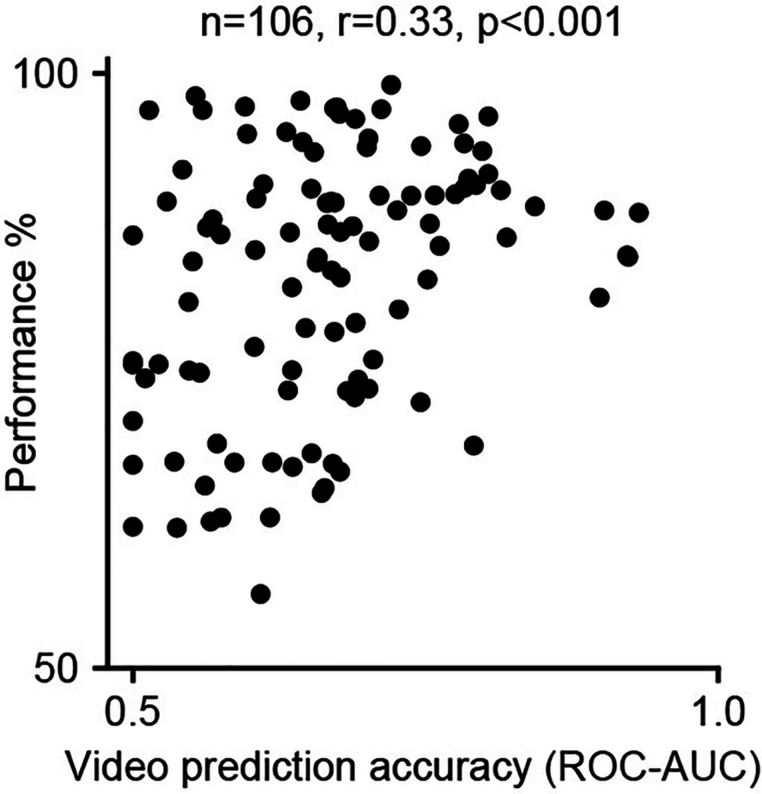
Correlation between predictability of behavior from video and task performance. The x axis corresponds to the mean ROC-AUC for predicting behavior directly from video during the delay epoch. Each dot corresponds to a session. The y axis is the behavioral task performance of that session.

To visualize the nature of uninstructed movements, we first sorted trials according to prediction confidence. We found that in highly predictable trials, mice displayed mostly stereotypical patterns of behavior, but the specific movements were diverse across sessions and subjects. A subset of mice tended to have more uninstructed movements in lick-left trials (Supplementary Video 1), whereas another subset had stronger movements during lick-right trials (Supplementary Video 2). For some mice, the uninstructed movements were jaw or paw movements (Supplementary Video 1), whereas other mice exhibited only jaw movements (Supplementary Video 2). The behavior of individual mice varied across days. For example, one mouse remained static before the go cue in lick-left trials (Supplementary Video 2) but performed stereotypical swinging of the paw in lick-left trials of the next day (Supplementary Video 3). To allow more interpretable analysis of the movements that predicted choice, we repeated the choice prediction analysis using single markers, instead of embeddings of the full video. Despite the weaker predictive power of single markers (response epoch: marker: 0.88 ± 0.01 s.e.m., *n* = 106 sessions; embedding: 0.96 ± 0.00 s.e.m., *n* = 106, *P* < 1 × 10^−6^, *n* = 106 sessions), the heterogeneity of uninstructed trial-related movements across mice was still present (Fig. 6e, Calinski–Harabasz clustering score for clustering of within-animal points 12.57, compared with null model value of 1.05 ± 0.54 s.d.; higher scores correspond to stronger clustering, *P* < 0.001; Methods). For some animals, movements of the nose were more informative than movements of the jaw, whereas in other mice jaw movement was more informative than nose movements (Fig. 6e). Interestingly, we found that such task-related preparatory movements during sample and delay epochs were positively correlated with the animal’s performance on a given session (*r* = 0.33, *n* = 106 sessions, *P* < 0.001; Extended Data Fig. 7 and Methods). These analyses reveal idiosyncratic patterns of uninstructed movements in individual mice that predict choice behavior.

### Activity related to uninstructed movement and decision-making

**Fig. 7:**
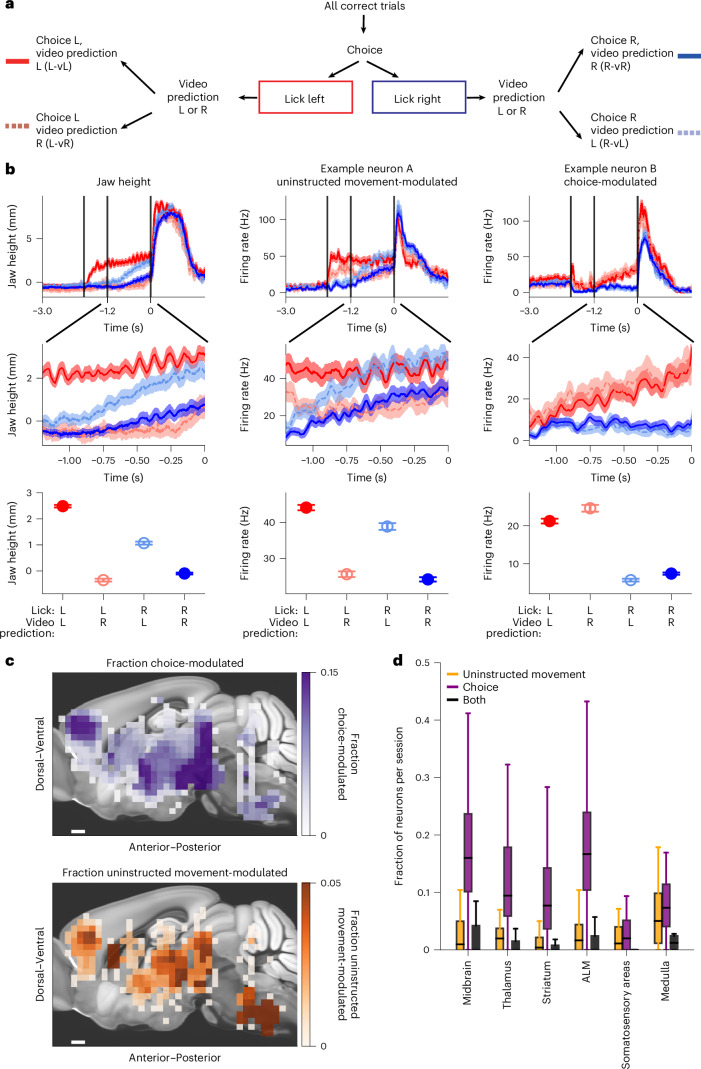
Single-trial analysis of movement and spike rate reveals neurons modulated by choice and movement. **a**, Schematic of analysis. Correct trials were split into lick-left and lick-right trials. Each of these sets of trials corresponding to a choice was further broken into two groups based on the prediction of behavior from video, yielding four groups of trials corresponding to the choice contingency and value of single-trial video prediction (Methods). **b**, Analysis schematic as applied to two example neurons (center, right) and jaw marker (left) within a single session. Left: jaw marker position (height) during entire trial (top) and magnified on the delay epoch (middle). Lines correspond to the mean of jaw height across the four trial groups. Color indicates trial type and line style (solid or dashed) corresponds to the video-prediction-based contingency. Shaded area corresponds to the s.e.m. across trials. Center: same data as in top, magnified on the delay epoch. Bottom: mean jaw height during the delay epoch split into the choice and video prediction groups. Color indicates choice and line style type (solid or dashed) indicates agreement between choice and video prediction contingency. Error bars correspond to the s.e.m. across trials (the numbers of trials in each group are: L-vL, 111; L-vR, 70; R-vL, 63; R-vR, 104). Middle: firing rate of example neuron analyzed according to groups defined in **a**. Top: firing rate of example neuron during the entire trial divided into the same four groups as **a**. Color indicates trial type and line style indicates agreement between choice and video prediction contingency. Center: same data as in top, magnified on the delay epoch. Bottom: average firing rate during the delay epoch split into the trial type and video prediction groups. Color indicates trial type and line style type agreement between choice and video prediction contingency. Error bars correspond to the s.e.m. across trials. Neuron is modulated mainly by uninstructed movements. Right: same format as in middle column for a different example neuron that is modulated mostly by choice. **c**, Brain-wide spatial map of the fraction of neurons modulated by choice (top) and modulated by uninstructed movement (bottom). Each voxel is 300 µm squared in the sagittal plane and spans the brain in the third direction. Color corresponds to the fraction of choice-modulated neurons (top) and uninstructed movement-modulated neurons (bottom) within that voxel. For visualization, a 3 × 3-voxel median filter was applied on the heatmap. Scale bar: 0.5 mm. **d**, Fraction of neurons modulated by choice, uninstructed movement or both for six brain regions. The midline of the boxes represents median, the box edges are interquartile range and the whiskers are 1.5 × interquartile range for the fractions in individual sessions (*n* = 87 sessions). Neurons were classified into choice- or uninstructed movement-modulated according to differences in prediction AUC across the four trial contingencies (Methods). L, left; R, right.

**Extended Data Fig. 8:**
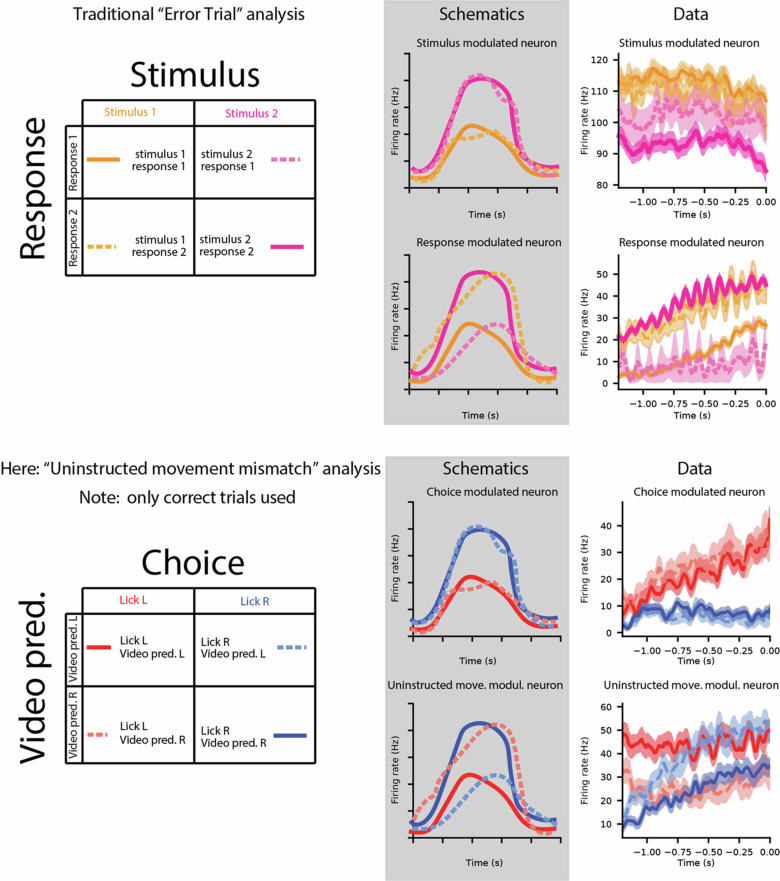
Schematic of trial contingency based single neuronal tuning analyses. On the top the traditional response-stimulus analysis is outlined that uses error trials to differentiate between tuning to response versus stimulus for individual neurons. In the middle two example schematics are shown for stimulus (top) and response (bottom) tuning; and two example neurons are shown on the right. The traces correspond to trial averaged mean traces during the delay epoch, and the shaded areas correspond to one standard deviation. The bottom half outlines the video-prediction-based analysis that uses single trial level choice predictions from videos to distinguish between modulation by choice versus uninstructed movements. The middle shows two example single-neuron schematics for choice (top) and uninstructed (bottom) movement modulation. On the right, two example neurons are shown. Traces correspond to trial averaged mean traces during the delay epoch, and the shaded areas correspond to one standard deviation.

Given that uninstructed movements can predict future behavior, we explored the interplay between encoding of uninstructed movements and choice-related neural activity. When movements can predict future choice, movement encoding is entangled with choice encoding. Analogously to the way error trials are often used to differentiate stimulus encoding from action encoding, in which the task instruction and action are dissociated^3,34^, we used trials where the behavioral choice and the choice predicted from the uninstructed movements during the delay epoch were in disagreement to dissociate choice and movement encoding (see Extended Data Fig. 8 for schematic). We analyzed correct trials only and separately divided lick-left (L) and lick-right (R) trials into two groups based on the prediction of the video-based classifier (predicted lick-left (vL) and predicted lick-right (vR) for each trial type; Fig. 7a), thus obtaining four trial contingencies (L-vL, L-vR, R-vR, R-vL; Methods). When considering single-neuron tuning properties, in trials where choice and video prediction match (L-vL and R-vR), choice tuning and movement tuning are confounded. In contrast, trials with mismatches between choice and video prediction enable us to potentially distinguish between tuning to choice versus movements (L-vR and R-vL).

We analyzed each neuron for differences of spike rates across these four groups of trials. Some neurons’ spike rates were strongly modulated by choice and did not change across different video predictions (that is, same response in R-vR and R-vL, and same response in L-vR and L-vL). We refer to these as choice-modulated neurons (Fig. 7b). This was not due to the video decoders picking up only on minute movements that would be unlikely to drive neural modulation, as we observed strong differences in multiple behavioral features across the video prediction groups (Fig. 7b). For instance, jaw height was significantly different across same choice groups with different video predictions in most sessions (Fig. 7b, significantly differentiated R-vR versus R-vL in 56 of 80 sessions and in 56 of 80 sessions for L-vR versus L-vL, *P* < 0.05; only sessions with moderate and higher behavioral predictability were chosen for this analysis, defined as AUC > 0.6; Methods). Indeed, other neurons’ spike rates were strongly modulated by the grouping of video prediction even when conditioned for the choice (same response in R-vR and L-vR, and same response in R-vL and L-vL). We refer to these as uninstructed movement-modulated neurons (Fig. 7b). Some neurons were modulated both by choice and uninstructed movement.

**Extended Data Fig. 9:**
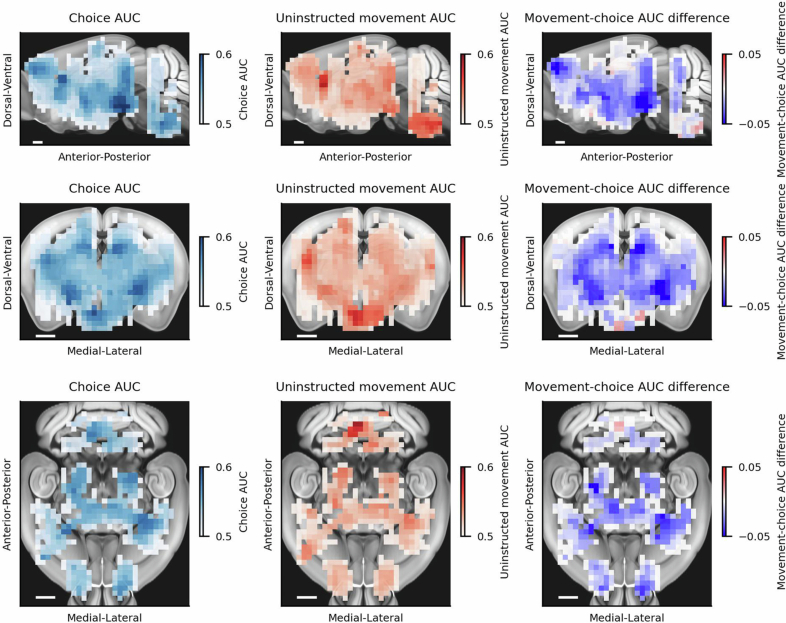
Spatial distribution of choice and movement prediction single neuron AUC. The heatmaps show the distribution of choice prediction AUC (left column), uninstructed movement related AUC (middle column) and the difference between the two (right column) in the sagittal plane (top row), coronal plane (middle row), horizontal plane (bottom row). Each voxel is 300 µm squared in the respective projection plane and spans the brain in the third direction. Color corresponds to the average AUC of neurons within that voxel for choice (left column), uninstructed movement (middle column) and the difference of these (right column). For visualization a 3x3 voxel median filter was applied on the heatmaps.

**Extended Data Fig. 10:**
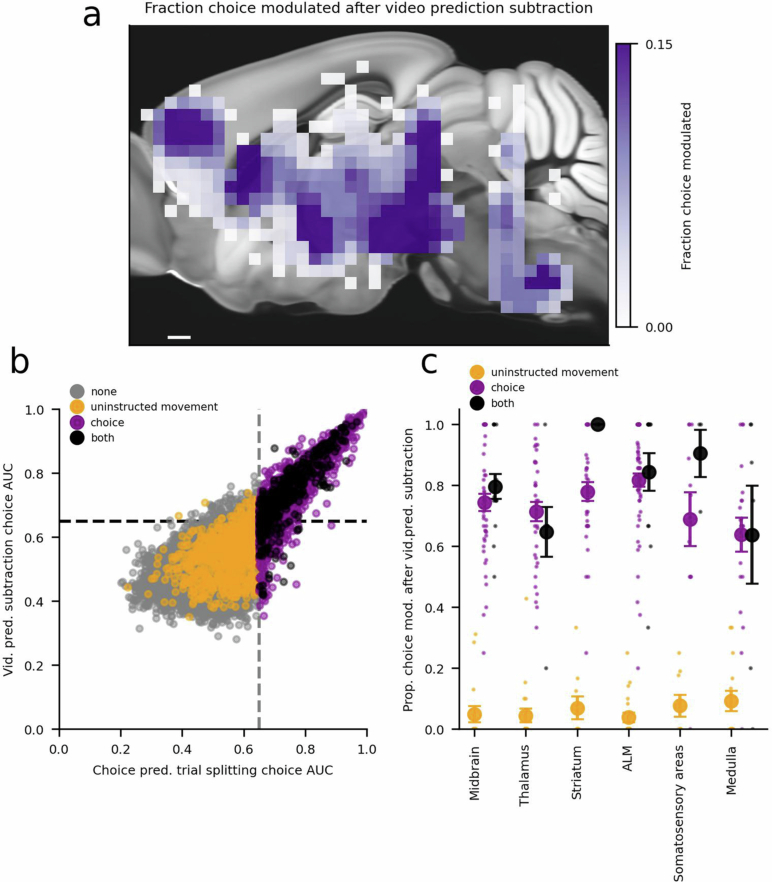
Comparison of methods for identifying choice modulated neurons. **a**. Proportion of choice modulated neurons in the sagittal plane. Each voxel is 300 µm squared in the sagittal plane and spans the brain in the third direction. Color corresponds to the fraction of choice modulated neurons in each voxel. For visualization a 3x3 voxel median filter was applied. **b**. Scatter plot for the choice AUC obtained by grouping trials based on video-based choice prediction (x-axis) and subtraction of movement related neural activity predicted by videos (y-axis). Colors correspond to modulation of neurons according to trial grouping-based AUC (same as in Fig. 7). Dashed lines show the AUC threshold for choice modulation. **c**. Proportion of neurons categorized as choice modulated based on the choice AUC obtained after subtraction of movement related neural activity. Colors correspond to modulation of neurons according to video-prediction-based trial grouping AUC (same as in Fig. 7). Large markers correspond to mean of the fractions across sessions, error bars show SEM. Overlaid small markers show the fractions in individual sessions.

The relative proportions of choice-modulated and uninstructed movement-modulated neurons varied across the brain (Fig. 7c,d and Extended Data Fig. 9; AUC test against spatial uniformity, *P* < 0.001), but the two types of neurons were spatially intermingled within each area. The relative strength of modulation also varied across brain regions (Extended Data Fig. 9; defined as the difference in AUC for choice and uninstructed movements, test against spatial uniformity, *P* < 0.001). ALM and midbrain neurons were more likely to be choice-modulated than medulla neurons (proportion of choice- to uninstructed movement-modulated neurons, ALM 4.8 versus medulla 1.2, *P* < 0.001; midbrain 4.3 versus medulla 1.2, *P* < 0.001, binomial test with Bonferroni correction). This is consistent with the reported roles of ALM and subregions of midbrain in decision-making tasks^3,30,35,42–44^ and that of medulla in low-level motor control^39^. We also tested a different approach, predicting and then subtracting movement-related neural activity, followed by reassessing choice selectivity, and found largely consistent results (Extended Data Fig. 10). In summary, our analysis of the relationship between video-based behavior prediction and spike rates allowed us to disentangle neural coding of movement from decision-related activity, which revealed clear differences in encoding across different brain areas and identified regions of interest for choice computation.

## Discussion

We analyzed movement-related activity across the brain during a decision-making task. We present multiple methods to relate neural activity to movements captured by behavior videos, with less interpretable nonlinear methods yielding superior predictions in terms of explained variance. Movement-related signals were pervasive across the brain, but their strength differed across areas. Analysis of activity following movement versus leading movement revealed a rich structure of sensory versus motor processing between and within brain regions. Choice-related uninstructed movements were common but varied greatly across animals and sessions. We used single-trial analysis to tease apart activity modulation by uninstructed movements versus coding of choice.

Movement-related neural activity has been investigated in multiple brain regions and by multiple methods^17,20,26,45–47^. Although differences between brain areas have been previously reported^17,20,47^, we provide a comprehensive characterization of movement-related activity across the brain in a single behavior. The temporal resolution of electrophysiological recordings allowed us to dissect neural activity related to motor versus sensory encoding, and, in anatomical structures known for sensory or motor processing, the overall organization agreed with expectations. At the same time, our analysis reveals rich encoding of movement within each area. The neural activity of intermingled neurons can lead or lag movement. The observed large spread suggests broadly distributed, closed sensorimotor loops, with most brain areas participating in controlling movement and responding to movement. Resolving whether the activity leading versus lagging movement truly reflects reafferent versus efferent signals will require experimental manipulations. For example, in the rodent whisker system, lesion of the infraorbital nerve can abolish reafferent signals from the vibrissa, while leaving efferent signals relatively intact^10^. However, such experimental manipulation has been done only in limited cases. Our analysis can provide a first-order localization of relevant signals to guide further manipulation experiments.

When studying neural computations underlying cognitive processes, we may wish to disentangle these forms of encoding from activity related to uninstructed movement. Previous studies have tackled this question in multiple ways. For example, one can try to regress-out the movement-related part of neural activity^17,30,45^, or define different subspaces in relation with movements^46^. Here, we used single-trial-level predictions of choice from videos to identify trials where animals’ uninstructed movements differed from those most characteristic of a given upcoming choice. While earlier work relying on encoding models has found that a large portion of trial-by-trial variance can be attributed to movements^17,42^, we have shown that in multiple regions upcoming choice is also encoded independently of movements. We were able to uncover significant differences between areas in the prevalence of neurons tuned to the choice- versus task-related uninstructed movements. Our approach, as all decoder-based approaches, might suffer from the choice of the specific predictors used, both in terms of their ability to account for different forms of movement as well as their ability to generalize across variations of similar movements, and therefore the question of whether we captured all the relevant uninstructed movements remains open. We believe that this form of video-prediction-based dissection of modulation is complimentary to other approaches and can be broadly useful to disentangle different trial-related forms of encoding.

Uninstructed movement could be part of an animal’s strategy in solving the cognitive task^48^ or reflect the state of the animal or other ongoing processes^26,45^. Although video-based choice prediction was positively correlated with the performance of the animal, the explanatory power of the correlation was weak, and multiple animals performed the task at a high level without engaging in choice-related uninstructed movements. On one hand, this could stem from weaknesses in the choice-related movement prediction methods or from the animal engaging in movements that are outside of the camera’s field-of-view, such as posture and hind-leg movements^17^. On the other hand, some animals are likely to be able to solve this cognitive task without relying on overt, uninstructed movements. One way to explore this question further is to study task-related uninstructed movements in more complex tasks, as one could expect that if uninstructed, but task-specific, movements are a useful strategy, or even a necessary strategy, then task-related uninstructed movements should be more prevalent in more demanding tasks.

Our finding that direct end-to-end methods outperform other methods was surprising given the large number of parameters to be fit in that approach, the limited number of trials available from training and the known variability in single-trial responses, all of which could have reasonably led to overfitting. This suggests that for studies focusing on questions such as dissection of movement-related responses, in which the main goal is to identify (and then potentially subtract) movement-related activity, end-to-end models would be an appropriate tool. However, the key disadvantage of these models is poor interpretability. If the goal is to understand the different aspects of movements neurons are tuned to, we believe the autoencoder-based embedding space approach is more favorable. Unlike marker-based methods that require specific body parts to be defined in advance for tracking, the autoencoder extracts the aspects of movement that are then tracked in the embedding space directly from data, which can be beneficial since it is difficult to put in place reasonable priors for the range of movements that might modulate neural activity. Additionally, the embedding space can be trained once and then allows for many different analyses to be performed on top of it and experimented with, while incurring relatively little additional computational cost. This key advantage may outweigh the additional predictive power gained by using the end-to-end approach.

What is the importance of widespread and intermingled efferent and reafferent signals? The existence of motor signals could be the result of an explicit computational strategy to supply these signals as they are necessary for the area’s function. For instance, an area involved in active sensing likely needs information about the motor commands, both as they could influence the active sensing strategy and because they may affect the sensory apparatus^13,49,50^. However, identifying specific signals does not by itself indicate their functional necessity for an area’s computations. Given the dense local and inter-regional connectivity, perfect filtering of signals unrelated to a region’s function could require overly complex and inefficient gating mechanisms. An alternative computational strategy is to isolate the dynamics needed for an area’s computations from irrelevant signals, for instance, by organizing them into distinct subspaces in activity space^51–53^. To test these possibilities, an area’s dynamics could be perturbed specifically along directions associated with specific information. This could be accomplished by simultaneous imaging and online targeting of perturbations^54,55^.

## Methods

### Data collection and preprocessing

We analyzed a publicly available dataset published in refs. ^30,31^. This study is based on data from 28 mice, including 25 VGAT-ChR2-EYFP (The Jackson Laboratory, JAX no. 014548), one C57BL/6J (JAX no. 000664), one Sst-IRES-Cre (JAX no. 013044) crossed with reporter mouse Ai32 (JAX no. 024109) and one Emx1-IRES-Cre (JAX no. 005628) crossed with R26-LNL-GtACR1-Fred-Kv2.1 reporter mouse (JAX no. 033089). The mice were 3–7 months old at the time of recording. All procedures were in accordance with protocols approved by the Janelia Research Campus Institutional Animal Care and Use Committee.

The data were obtained from Neuropixels probes^33^ used to record extracellular activity in multiple regions of the mouse brain. To perform spike-sorting, we used Kilosort^56^ with a custom quality control pipeline outlined in a whitepaper^57^ (10.25378/janelia.24066108.v1). We then binned spikes into firing rates with a bin width of 40 ms and a stride of 3.4 ms.

In addition to neural activity, we recorded high-speed (300 Hz) multiview video of the face, paws and body of the mouse using complementary metal-oxide semiconductor cameras (CM3-U3-13Y3M, FLIR) under infrared (940 nm light emitting diode) light, with 4–12-mm focal length lenses (12VM412ASIR, Tamron), achieving a pixel resolution of 71 µm. For analysis, only the side-view frames were used. We used DeepLabCut^24^ to obtain the positions of the jaw, paws and tongue. We refer to these positions as markers. We manually labeled about 2,800 frames, and trained the model using this software, and we used the same model across all sessions. Although these markers were mostly reliable, we found outliers that harmed the prediction of firing rates or animal behavior. We identified outliers by a five-sigma threshold on velocity across frames and imputed outliers from nearby frames. When the tongue was occluded while it was in the mouth, as was typically the case before the response epoch, we set the tongue position to its mean value. We note that other choices of specific data imputation method did not qualitatively affect the results.

Further details regarding the animals, behavior and data collection, including electrophysiology, video tracking, spike-sorting and histology, can be found in ref. ^30^.

### Convolutional autoencoder

The architecture of the convolutional autoencoder we used was similar to BehaveNet described in ref. ^22^. The encoder was composed of an initial convolutional layer, two residual blocks^58^ and two fully connected layers. The initial convolutional layer has 3 × 3 kernel size and 16 output channels and is followed by ReLU activation and a 2 × 2 max pooling. Each residual block was composed of four convolutional layers. Each convolutional layer has kernel size 3 and stride 1. The first residual block had 16 channels; and the second residual block increased the channel number to 32 in the first layer. Each convolutional layer is followed by ReLU activation and the second residual block ends with a max pooling with kernel size 4. The input image was resized into a 120 × 112 matrix. The output of the last convolutional layer was a vector with a length of 288. This output was then processed by the two fully connected layers (288 × 128 and 128 × 16) with ReLU activation between them yielding the output of the encoder, the embedding vector, with a length of 16. The decoder was a fully connected linear layer.

We trained and primarily used session-specific autoencoders. However, we also verified that one can train a session-independent encoder with session-dependent decoders. We note that decoders had to be session-dependent due to differences in overall position of the mouse, experimental components and background. In other words, during training, all frames are fed into the same encoder but will then go to different decoders depending on which session they were extracted from. We verified that training a session-independent autoencoder with 40 sessions yielded similar performance and qualitatively similar analysis results to a session-dependent decoder. We also verified that the encoder can then generalize to sessions that the encoder has never seen before.

### End-to-end learning framework

In the end-to-end learning framework, we trained deep neural networks to directly predict neural firing rates. For each session with each brain region, we trained a neural network. The network was composed of three residual blocks^58^ and a final linear output layer. Each residual block was composed of four convolutional layers, and each convolutional layer was followed by a two-dimensional batch normalization with epsilon 1 × 10^−5^ and momentum 0.1. The first convolutional layer of a residual block had kernel size 1 and stride 1; the latter three had 3 × 3 kernel size and stride 1. The output of the first residual block had 16 channels, and the output of the other two blocks had 32 channels. Each residual block was followed by a two-dimensional max pooling with a kernel size of 4 and a stride of 4 for down sampling. After each batch normalization or max pooling, a ReLU activation was applied. After the three residual blocks and the max poolings, the output was a vector of length 160. A linear output layer was connected to the end of the last residual block with output size equal to the number of neurons to be predicted in the session.

### Prediction of neural activity using embedding or markers

When predicting neural activity at time *t* from embedding vectors or marker positions, we took a 5-frame window of the video and collected the respective features at *t* − 6.8 ms, *t* − 3.4 ms, *t*, *t* + 3.4 ms and *t* + 6.8 ms and concatenated these vectors, obtaining a single feature vector that was 80-dimensional for the embedding-based approach (16 latent dimensions times the 5 neighboring time points) and 15-dimensional for the marker-based approach (3 markers times the 5 time points). We then used L2 regularized linear regression (ridge-regression) to predict neural activity at time *t*. The regularization parameter was obtained through fivefold nested cross-validation. Neurons with low firing rates (below 2 Hz) were excluded from analyses; the results were not sensitive to the exact value of this threshold.

### Trial selection and cross-validation

The dataset contains trials with photoinhibition, water administration regardless of the animals’ choice (free water trials), early licks and trials where the animal ignores the lick-spouts. These were excluded from all analyses.

For the analyses comparing the three methods (Fig. 2 and Extended Data Figs. 2 and 3), we used a single, random train-test split, with balancing of the ratio of lick-left, lick-right and correct versus error trials in the training and test splits. The test split was 64 trials for all sessions, and the same splits were kept across methods.

For analyses involving only the embedding-based methods (Figs. 3–5 and Extended Data Figs. 4–6), we used fivefold cross-validation, stratified with regards to licking direction and correctness across the folds.

For analyses involving behavioral prediction (Figs. 6 and 7 and Extended Data Figs. 7 and 9), we selected only correct trials and used 20-fold stratified cross-validation.

#### Thalamic nuclei

For Fig. 3 and Extended Data Fig. 4 the following subregion definitions were used, based on the Allen ontology: CN, central lateral nucleus; central medial nucleus; MD, mediodorsal nucleus of thalamus; PC, paracentral nucleus; PO, posterior complex of the thalamus; VAL, ventral anterior-lateral complex of the thalamus; VM, ventral medial nucleus of the thalamus; VP, ventral posterior complex of the thalamus; ventral posterolateral nucleus of the thalamus, parvicellular part; ventral posteromedial nucleus of the thalamus; ventral posteromedial nucleus of the thalamus, parvicellular part.

### Epoch-averaged explained variance

To calculate epoch average explained variance we first calculated the explained variance for each time point and test-fold separately. Then, we rectified the explained variance scores by setting all negative values to 0. After this, the folds were averaged. Finally, we averaged the time points within the relevant epoch (sample, delay or response). To avoid edge effects and compare the same time points (for neural activity) across all time-shifts, we excluded the first and last 150 ms of each epoch when taking the average. We filtered out neurons with very low explained variances (below 0.01); the results were not sensitive to the exact value of this threshold.

### Identifying neurons with reliable firing patterns and poor video prediction

To identify neurons with highly reliable spiking patterns across trials but poor predictions from behavioral videos (as shown in Extended Data Fig. 1), we selected neurons with low epoch-averaged explained variance (embedding-based response epoch-explained variance < 0.1), with high correlation between the single-trial firing rate traces and the trial-averaged firing rate pattern (correlation > 0.4) and with low trial-by-trial variability (average across all time points of instantaneous trial-to-trial firing rate variance < 100 s^−2^). We have found a total of 196 such neurons in the whole dataset. The exact value of these thresholds does not change the qualitative type or the approximate proportion of these neurons found in the dataset.

### Analyzing optimal time-offsets

When analyzing the optimal time-shift between video and neural activity, we shift the video by tau (*τ*) in time (multiples of 6.8 ms which corresponds to two frames). Then we repeat the same analysis that we did when predicting neural activity using embedding vectors for a large set of possible time-shifts (−102 ≤ *τ* ≤ 102 ms): we take the features corresponding to five neighboring frames at *t* + *τ* − 6.8 ms, *t* + *τ* − 3.4 ms, *t* + *τ*, *t* + *τ* + 3.4 ms, *t* + *τ* + 6.8 ms; predict neural activity using ridge-regression for every time point separately; and calculate the epoch-averaged explained variance for each possible *τ* time-shift. The optimal time-shift is the one that maximizes the explained variance τ* = max_*τ*(*R*^2^(*τ*)). Since some neurons’ explained variance curves as function of time-offset were flat, we kept only neurons with well-defined peaks (max(*R*^2^) ≥ 1.2mean(*R*^2^), where mean(*R*^2^) is the mean explained variance across all time-shifts); the results were not sensitive to the exact value of this threshold.

To assess the significance of a neuron’s time-offset (Fig. 5), we used the explained variances from the different cross-validation folds and compared the optimal time shifts coming from the different folds against zero, using a *t*-test to obtain a *P* value for each neuron. Significance was established through Benjamini–Hochberg false discovery rate control, with rate parameter *Q* = 0.05.

### Defining choice- and uninstructed movement-modulated neurons using video-based choice decoder

To define choice- and uninstructed movement-modulated neurons (Fig. 7 and Extended Data Fig. 9), we used all correct trials and split them into four groups based on licking direction and the delay epoch video prediction (obtained as described above). Sessions with fewer than 20 trials in any of the four groups were excluded. We used regularized logistic regression either to predict choice while conditioning for video prediction or to predict uninstructed movement type (video prediction) while conditioning for choice from average firing rates of single neurons during the delay epoch. The prediction was characterized by ROC AUC on the test-fold and the two conditions were averaged in each case, yielding a single AUC for choice prediction and another AUC for uninstructed movement prediction for each neuron. The final AUC for each neuron is the average of the test AUC across all test-folds. The regularization parameter was found through nested leave-one-out cross-validation.

A neuron was then grouped as choice- versus uninstructed movement-modulated if the relevant AUC exceeded 0.65; note that some neurons have AUC higher than this threshold for both variables.

### Defining choice-modulated neurons after subtraction of movement-related activity

To define neurons modulated by choice but unaffected by any uninstructed movements (Extended Data Fig. 10), we first subtracted the predicted per-timepoint neural activity based on the embedding method of each neuron. We then used regularized logistic regression to predict choice from single-neuron residual activity using fivefold cross-validation, with nested cross-validation to find the regularization parameter. Each neuron’s choice decoding AUC is the average test-fold AUC across all cross-validation folds. To classify a neuron as choice-modulated we used the same threshold as for the other method, AUC > 0.65.

### Calinski–Harabasz clustering score

The Calinski–Harabasz index, also known as the variance ratio criterion, is the ratio of the sum of between-clusters dispersion of a measured feature to the inter-cluster dispersion for all clusters. Higher scores indicate stronger clustering. To obtain a reference null-distribution for the Calinski–Harabasz score, we randomly assign the labels (mice) to each data point, and then calculate the score, repeating the random process 1,000 times. We assess significance by comparison to this null distribution.

We used this clustering score two times. In Fig. 6d, we take the AUC from the embedding framework as features and mouse identity as cluster labels. In Fig. 6e, AUCs from the jaw and the nose markers were used as features, and mouse identity was used as cluster labels. We note that we used only sessions where the trial type was predictable from either jaw or nose (AUC equal to or higher than 0.65) and only mice that had two or more of such predictable sessions.

### Testing against spatial discontinuity with nearest-neighbor explained variance difference distribution

To test whether spatially closer neurons have smaller difference in their explained variance than expected by chance within thalamus (Fig. 3e), we first identified neurons within each nucleus and calculated the cumulative distribution function for the explained variance difference between nearest neighbors. As a control we randomly shuffled the neurons’ explained variance values within each nucleus separately and calculated the cumulative distribution function for the explained variance difference in the shuffled sample. We repeated the shuffle 1,000 times to establish a null-distribution and bootstrap *P* value.

### Test against spatial uniformity

To test against spatial uniformity (Figs. 3 and 7 and Extended Data Fig. 9) of a given scalar statistic (explained variance, single-neuron AUC) given the fixed sampling provided by the CCF coordinates, we split the brain into cubic voxels (0.2 mm cubed for Fig. 3 and 0.5 mm cubed for Fig. 7 and Extended Data Fig. 9, but the results do not depend on the specific choice of this size) and grouped neurons based on these voxels. We then calculated an *F*-statistic for this grouping using one-way *F*-test. As a control we randomly shuffled the scalar statistics of interest (explained variance, single-neuron AUC) across neurons while keeping the CCF coordinates fixed; we used the same cubic voxels as before to group the shuffled neurons and calculated an *F*-statistic for this shuffle control using one-way *F*-test. We repeated the shuffle 10,000 times to establish a null-distribution and bootstrap *P* value.

### Data pooling

The registration of individual animals into a common reference frame (CCF v.3) allowed us to pool data across animals when it was necessary to combine neurons across animals (Figs. 2a, 3–5 and 7c,d). All analyses relating movement to neural activity were first carried out on a session-by-session basis and the pooling happened only on the level of single-neuron variables (for example, explained variance, best time-offset, AUC) with uniform weights across sessions.

### Reporting summary

Further information on research design is available in the Nature Portfolio Reporting Summary linked to this article.

## Online content

Any methods, additional references, Nature Portfolio reporting summaries, source data, extended data, supplementary information, acknowledgements, peer review information; details of author contributions and competing interests; and statements of data and code availability are available at 10.1038/s41593-025-02114-x.

## Supplementary information

Supplementary Information Supplementary Discussion, Table 1 and Figs. 1 and 2.

Reporting Summary

Supplementary Video 1 Example left and right trials with uninstructed movements during the sample and delay epochs. Animal with paw movements on left lick trials.

Supplementary Video 2 Example left and right trials with uninstructed movements during the sample and delay epochs. Animal with jaw movements on right lick trials.

Supplementary Video 3 Example left and right trials with uninstructed movements during the sample and delay epochs. Animal develops paw movements in left lick trials compared to session on a previous day in Supp Video 2.

## Data Availability

The data used for this study are publicly available via the DANDI archive at 10.48324/dandi.000363/0.230822.0128 (ref. ^31^).

## Code availability

All analyses were performed using custom Python code. Code is publicly available via GitHub at https://github.com/druckmann-lab/MapVideoAnalysis (ref. ^59^).
