# Supplementary material for: Brain-wide analysis reveals movement encoding structured across and within brain areas
Source: Nat Neurosci. 2025 Nov 18;29(1):147–58. doi: 10.1038/s41593-025-02114-x (PMC12779545; doi:10.1038/s41593-025-02114-x)
Supplement: Supplementary file 1 — Supplementary Discussion, Table 1 and Figs. 1 and 2. [file 41593_2025_2114_MOESM1_ESM.pdf]

nature neuroscience

Supplementary information

https://doi.org/10.1038/s41593-025-02114-x

Brain-wide analysis reveals movement encoding structured across and within brain areas

In the format provided by the authors and unedited

Supplementary Discussion

Session-independent auto-encoder

We have described the session-independent auto-encoder in the method section. Here, we show the results of this method. For each of the 22 mice, we take 1 or 2 sessions to the training set, and thus we take 40 sessions to the training set in total, and we call the other 60 sessions the hold-out set. Then, we use the embedding vectors to predict the firing rates through ridge regression, in the same way we did for the embedding from the session-dependent version. In both training set and hold-out set, the session-independent auto-encoder's performance is much better than that of using the markers (in the training sessions exp. var. difference 18.2% +/- 1.2% sem, n=122 recordings, $p<0.001$. In the hold-out sessions difference 16.5% +/- 1.1% sem, n=192 recordings, $p<0.001$), though it's slightly worse than that of the session-dependent (in the training sessions exp. var. difference 3.6% +/- 1.1% sem, n=122 recordings, $p<0.001$. In the hold-out sessions, difference 12.8% +/- 3.0% sem, n=192 recordings, $p<0.001$). This result demonstrates that the encoder learns semantic information of the mice's behavior and can generalize to the sessions that the model hasn't seen in the training process. This method also helps researchers get embedding vectors in a very efficient way, as they will only need to train one model for all the sessions.

Supplementary Table 1: Thalamic nuclei

Thalamic sub-regions were defined according to the Allen reference atlas annotations. The association between the labels used in Figure 3 and annotations is given in the following table:

Label	Associated annotations
CN	Central lateral nucleus; Central medial nucleus
MD	Mediodorsal nucleus of thalamus
PC	Paracentral nucleus
PO	Posterior complex of the thalamus
VAL	Ventral anterior-lateral complex of the thalamus
VM	Ventral medial nucleus of the thalamus
VP	Ventral posterolateral nucleus of the thalamus; Ventral posterolateral nucleus of the thalamus, parvicellular part; Ventral posteromedial nucleus of the thalamus; Ventral posteromedial nucleus of the thalamus, parvicellular part

Supplementary Figures

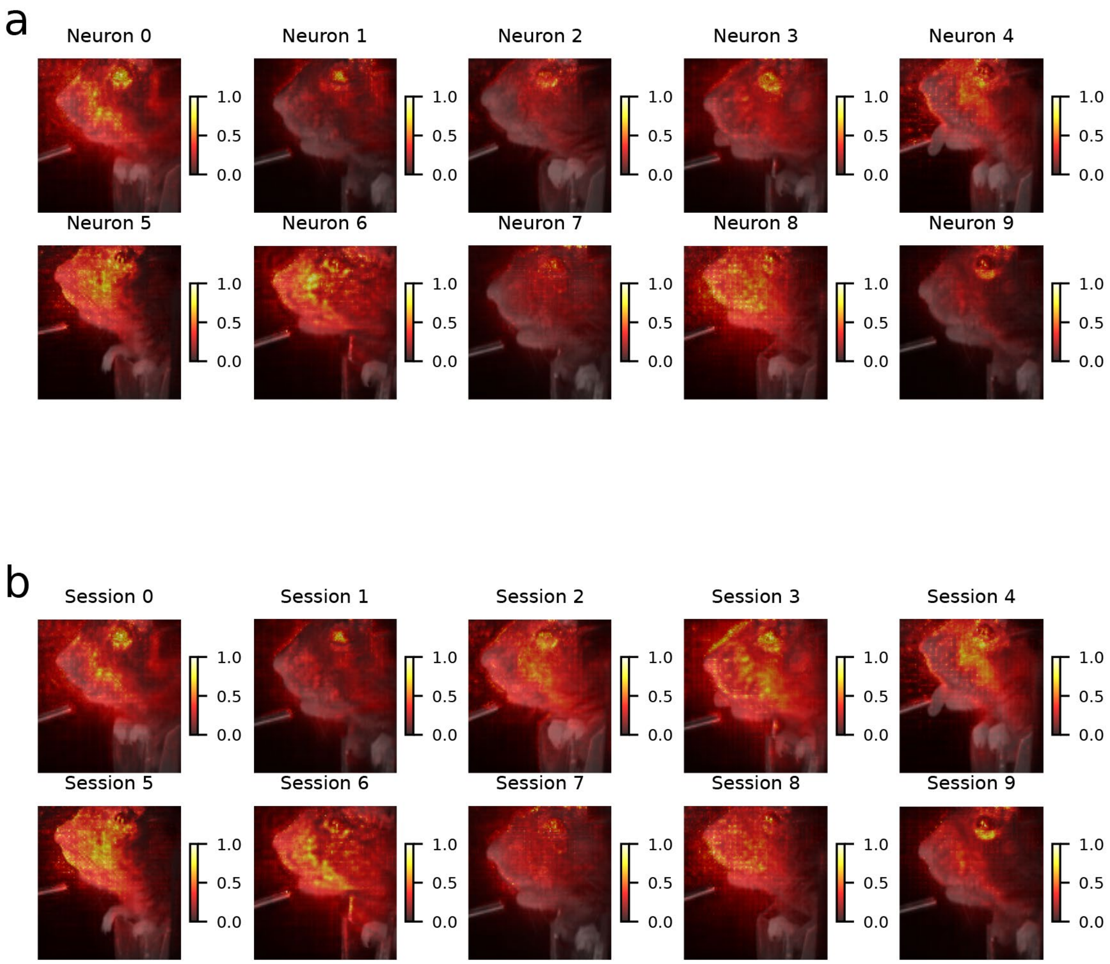

Supplementary Figure 1. Single neuron and single session saliency maps for the end-to-end method. a. Normalized saliency maps derived through gradient based backpropagation for the 10 neurons which showed the largest improvement with the end-to-end method over the embedding- and marker-based approaches. **b.** Saliency maps averaged for all neurons within the sessions that contain the neurons in a. Backgrounds are an example frame from the relevant session and colors represent saliency of each pixel normalized to 1.

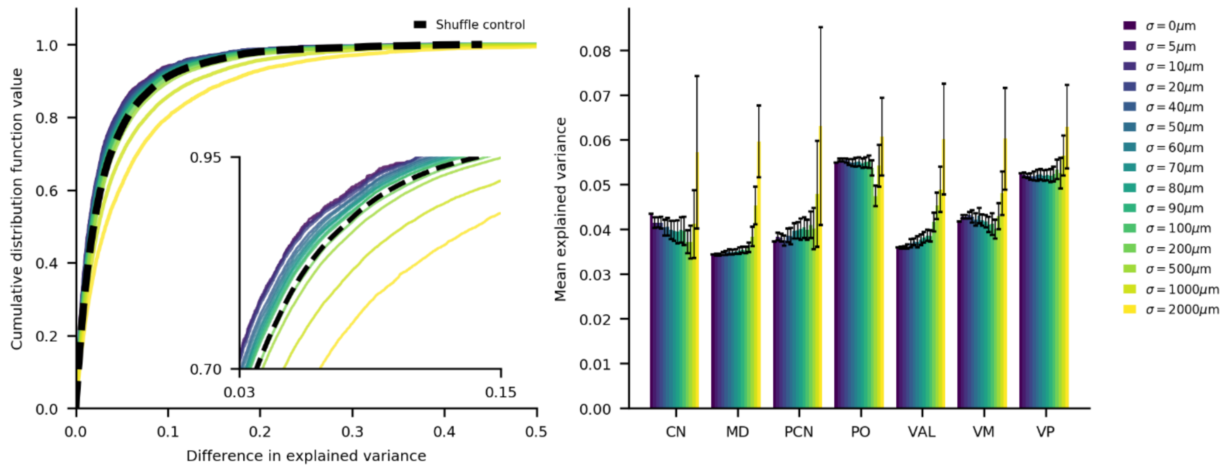

Supplementary Figure 2. Control for spatial localization errors in subregion analysis by spatial noise injection. On the left, the cumulative distribution function value is shown for the difference in explained variance for neurons within the thalamus as in Fig. 3e. Solid lines correspond to average cdf extracted from data across 20 random noise injections; colors correspond to the variance of 3D gaussian noise. Black dashed line corresponds to zero added noise shuffle control. Differences between shuffle and data are statistically significant up to the injection of Gaussian noise with 100 μm variance (in all three directions) with p=0.003 (comparison against shuffle control, for details see Methods). On the right, the average explained variance of each subregion is shown. Number of neurons in each region are: CN n=235, MD n=1112, PCN n=268, PO n=600, VAL =457, VM n=221, VP n=651 The color of the bars corresponds to the variance of 3D gaussian noise. Error bars correspond to bootstrap variance.
